# When empathy leads to aggression: The effects of empathy on punitive attitudes towards aggressors

**DOI:** 10.1111/bjso.12907

**Published:** 2025-05-23

**Authors:** Célia F. Camara, Alejandra Sel, Paul H. P. Hanel

**Affiliations:** ^1^ Department of Psychology University of Essex Colchester UK

**Keywords:** aggression, bias, empathy, moral judgement, punishment

## Abstract

When witnessing aggression, individuals often empathize more with victims than with aggressors, which may bias their perceptions and interpretations of the transgressions. However, the mechanisms underlying these biases remain poorly understood. Through two experiments, we investigated whether people's decisions to condemn aggressors are influenced by their predisposition to sympathize with the victim and explored how negative sentiments towards the aggressor may influence these decisions. Further, we tested the moderating role of callous‐unemotional traits, hypothesizing that moral judgements and decisions to punish may differ among individuals who are less emotionally responsive, as they are less likely to sympathize with victims. Our findings revealed that greater empathy for victims intensified punitive attitudes towards aggressors, primarily mediated by participants' negative evaluations of the aggressor. Notably, such empathic inclinations were less prevalent among individuals with higher levels of callous‐unemotional traits, as reflected by their lower concern for victims and greater inclination towards harsh punishments. These results offer insights into how justice‐related attitudes may be shaped and potentially biased by individual differences in emotional responsiveness.

## INTRODUCTION

An examination of empathy affords a powerful site from which to rethink the fundamental commitments of social science broadly and the sociology of punishment specifically. (Brown, 2012, p. 383)

The exploration of human responses to perceived injustice reveals the complexities embedded within our collective moral fabric. For example, brutal aggressions towards innocent victims often elicit a shared sense of moral outrage that often leads to demands for severe punitive measures (Carlsmith & Darley, 2008). Such an inclination is rooted in the popular belief that punishment should mirror the severity of the suffering inflicted on victims (Bastian et al., 2011; Carlsmith et al., 2002), suggesting a bias in the extent to which individuals empathize with victims over perpetrators. In the present research, we investigate how such biases might be determined by the perceived intentionality of the transgression, while also exploring how judgements of the transgressor might further influence punitive attitudes. The goal is to gain insights into how individual's predispositions to selectively apply empathy towards victims over perpetrators might shape their reactions to perceived injustice.

### The empathic bias in moral judgement

Empathy has been broadly defined by its affective and cognitive facets, comprising the capacity to share and understand the subjective experience of others in reference to oneself (Decety, 2010). However, empathy can also refer to the capacity to feel concern about the welfare of others, which plays a key role in guiding moral behaviour (Decety et al., 2012; Decety & Jackson, 2004). Theoretically, it has been proposed that empathic concern prompts individuals to engage in charitable and altruistic behaviours (e.g. Batson et al., 1983; Underwood & Moore, 1982), as they are more prone to consider the well‐being of others. Empathic concern has also been proposed to guide moral decision‐making, helping internalize right from wrong by considering the perspectives, feelings and needs of others (Kohlberg, 1981; Piaget, 1932). Nevertheless, under specific circumstances, directing empathy towards some individuals may paradoxically diminish our empathy towards others (Simas et al., 2020). For example, people tend to show stronger empathy and helping intentions for ingroup members, which has been correlated with less empathetic responses towards those perceived as outgroup members (e.g. Cikara & Fiske, 2013; Tarrant et al., 2009, 2012; Vanman, 2016). Notably, this bias also seems to contribute to prejudice and discrimination against outgroups (Lalonde & Silverman, 1994; Tyler & Blader, 2003; Vanman, 2016).

In the context of aggression, empathic biases can look like taking the victim's side over the perpetrator's. By adopting the victim's perspective, individuals might engage in self‐referential cognitive processes, amplifying their sensitivity to the victim's suffering as well as a sense of injustice on the victim's behalf (Ames et al., 2008; Ruby & Decety, 2004). These reactions may in turn foster negative evaluations of the perpetrator, thereby influencing decisions to punish them (e.g. Lin et al., 2024). A recent study by Lu and McKeown (2018) provides some evidence for this. Using a Dictator Game task, the authors found that participants with higher levels of empathic concern were more predisposed to punish unfair outgroup members than they were to compensate ingroup members. Similarly, in an earlier study, participants' expressions of empathic anger on behalf of a victim of aggression significantly predicted their intentions to punish the perpetrator (Vitaglione & Barnett, 2003). These findings provide important insights as to how empathy, in some contexts, might indeed lead to aggression (Batson, 1997; see also Neuberg et al., 1997).

It is important to note, however, that the application of punishment as a penalty for an immoral action can be seen as a social response seeking to maintain societal harmony, and thus can be described as aggression with prosocial motives (Levy, 2022). At the same time, these prosocial motivations differ across individuals (Shichman & Weiss, 2022), meaning that the underlying nature as to why someone decides to punish a transgression might be subject to their inherent biases and perceptions.

### The psychology of punishment

Research in psychology shows that people's evaluation of transgressions is often shaped by the extent to which blame is ascribed to the transgressor (e.g. Feather, 1994; Feather & Dawson, 1998; Weiner, 1995). As such, the same act may be judged differently depending on the perceived intentions of the perpetrator, as demonstrated in studies using ‘moral sensitivity’ tasks (e.g. Baez et al., 2014, 2017; Decety et al., 2012; Santamaría‐García et al., 2017; Young & Saxe, 2008). In these tasks, participants are typically presented with scenarios where a transgression occurs either accidentally or intentionally. Findings consistently reveal that participants are more likely to morally condemn intentional transgressions compared to accidental ones (Baez et al., 2014; Bastian et al., 2011). Conversely, judgements of aggression may be more lenient when aggressors are perceived as not responsible for their actions or when the aggression is justified for a ‘greater good’ (Mikula, 2003).

This aligns with the Attribution of Blame model described by Shaver and Shaver (1985), which posits that assessments of injustice hinge on the degree of responsibility attributed to the perpetrator for violating someone else's entitlements without adequate justification. For example, while intentional harm to innocent victims is condemned and penalized (Mikula, 2003), aggressions towards violent offenders—often enforced through extreme forms of punishment such as capital penalties or castration—tend to be more justified and endorsed by the public (e.g. Bastian et al., 2013; Viki et al., 2012). This justification hinges on the idea that justice is implemented when punishments are proportional to the harm inflicted, with harsher punishments often perceived as effective strategies to both prevent the offender from reoffending as well as to discourage others from committing similar crimes. However, it should not be ignored that punishment may also be an emotional response to perceived injustice, which may drive individuals to advocate for (and potentially engage in) more severe measures for retaliation.

According to the Retributive Justice model proposed by Carlsmith et al. (2002), negative emotions arising from perceived injustice towards victims can lead to dehumanizing perpetrators (see also Darley & Pittman, 2003). This process may occur when perpetrators are seen as violating moral principles or social norms, justifying perceptions of them as less human and undeserving of basic rights (Haslam et al., 2005). Such judgements gain special relevance in legal contexts, where the severity of punishment for the wrongdoer is often based on the perceived intentionality of the committed crime (Bastian et al., 2011). This can lead to the endorsement of harsher punitive measures and reduced advocacy for the reform and reintegration of violent offenders, particularly in cases where perpetrators are perceived as inherently immoral or beyond redemption (Bastian et al., 2013; Harris & Rice, 2006; Osofsky et al., 2005). For instance, Viki et al. (2012) found that dehumanizing sex offenders positively correlated with the public's desire to exclude them from society, reduced support for their rehabilitation and increased endorsement of castration as a form of punishment (see also Bastian et al., 2013). These propositions raise important questions about the extent to which empathic biases and negative character judgements might influence moral reasoning and justice‐related decisions:

Hypothesis 1 We hypothesize that greater empathy towards victims will be associated with harsher punitive attitudes, especially when the harm is perceived as intentional (Baez et al., 2014; Bastian et al., 2011). We propose that this relationship will be mediated by the level of concern for the perpetrator, and the extent to which the perpetrator is perceived as ‘mean’. Specifically, we hypothesize that higher levels of empathy for victims will correlate with a decreased level of concern for perpetrators as well as an increased perception of the perpetrator's meanness, both of which are expected to be related to harsher punitive attitudes towards perpetrators (Bastian et al., 2013; Decety & Cowell, 2015; Viki et al., 2012).

### Individual differences in perceptions of injustice

Research suggests that people's responses to perceived injustice may vary depending on their emotional involvement when making moral judgements (Brown, 2012; Decety & Cowell, 2015; Mikula, 2003). Therefore, an individual's capacity (or willingness) to emotionally engage with others' experiences is key when examining their drive to retaliate on the behalf of others. Compelling examples can be found in the context of psychopathy. People with psychopathic tendencies are typically less sensitive to perceived injustice (Decety & Yoder, 2016). For example, individuals with psychopathy often engage in calculated and goal‐oriented aggression (e.g. using manipulation or deceit to achieve personal gain) even when fully aware that their actions cause harm to others (Gini et al., 2015). Similarly, studies have shown that psychopaths can correctly identify morally wrong actions but fail to show guilt or remorse for their wrongdoings (Koenigs et al., 2012). To put it simply, ‘(p)sychopaths know what is right or wrong, but simply don't care’ (Cima et al., 2010, p. 66).

Such disregard for others has been attributed to the expression of callous‐unemotional traits (Lockwood et al., 2013). Callous‐unemotional traits denote a cluster of personality characteristics marked by a lack of empathy, remorse and guilt, alongside shallow affect and a diminished sensitivity to others' emotional experiences (Frick et al., 2003). Individuals exhibiting these traits—even at subclinical levels—tend to approach moral dilemmas with a more detached and utilitarian perspective (Koenigs et al., 2012). Callous‐unemotional traits have also been linked to a greater tendency to justify or normalize aggressive behaviour, particularly in situations involving personal gain or social dominance (Barchia & Bussey, 2011; Gini et al., 2015). Such tendencies could shift moral judgements, potentially reducing feelings of guilt or remorse in the face of immoral actions and perpetuating harmful behaviours (Čehajić et al., 2009; Perren & Gutzwiller‐Helfenfinger, 2012).

That is not to say, however, that all individuals exhibiting callous‐unemotional traits are inherently immoral and aggressive (Campos et al., 2022). In fact, research on successful psychopathy indicates that some psychopathic traits may be advantageous in certain circumstances (Hall & Benning, 2006). For example, individuals with callous‐unemotional traits may be less influenced by emotional appeals when making moral judgements (Fragkaki et al., 2016; Vasconcelos et al., 2021). This reduced susceptibility to emotional distress, in turn, can be beneficial in professions that require emotional detachment, such as law enforcement (Dutton, 2012). From this perspective, one could argue that callous‐unemotional traits might reduce affective biases during punishment decision‐making, although no previous study has investigated this. We hypothesize the following:

Hypothesis 2 Extending *Hypothesis 1*'s prediction that greater concern for victims will correlate with harsher punitive judgements, we further hypothesize that individual differences in callous‐unemotional traits will attenuate this effect. Specifically, we propose that the positive relationship between victim‐focused concern and punishment severity will be significantly weaker at higher levels of callous‐unemotional traits, given that individuals with these traits are less likely to feel concern for victims (e.g Decety & Yoder, 2016). Additionally, we explore whether these hypothesized effects might influence punitive attitudes as well.

### Overview of studies

We conducted two studies to test our hypotheses. Study 1 required participants to evaluate scenarios depicting interpersonal harm, both intentional and accidental. Participants were asked to express their concerns for both the victim and the perpetrator, assess the perceived ‘meanness’ of the perpetrator, and determine appropriate levels of punishment. Study 2 replicated this task, adding a measure of participants' self‐reported levels of callous‐unemotional traits to explore how responses in the task could be influenced by the expression of these traits. Data and analysis code can be accessed at https://osf.io/bfmnt/?view_only=180a8ea9aea140b69f97af6b4b238ffc. Data were analysed using IBM SPSS Statistics for Windows, version 29.0 and *R*, version 4.0.0 (R Core Team, 2020). Study design and analysis were not preregistered.

## STUDY 1

### Method

#### Participants

A power analysis indicated that 111 participants were required to detect a small‐to‐medium effect size (*f* = 0.175) in a 3 × 2‐within‐subjects design with 95% power. A total of 126 participants were initially recruited at the University of Essex (UK). However, 15 participants did not complete the task, and one was excluded for failing attention checks, resulting in a final sample of 110 volunteers. The sample primarily consisted of university students aged 18–33 years (*M* = 21.69, *SD* = 2.77). The group included 76 women, 34 men, and one participant identifying as non‐binary/third gender. Ethnically, 61.3% identified as White, 21.6% as Asian/Asian British, 7.2% as Black, and 9.9% as belonging to multiple or other ethnic groups. Ethical approval was obtained from the first author's institution.

#### Experimental design and stimuli

**FIGURE 1:**
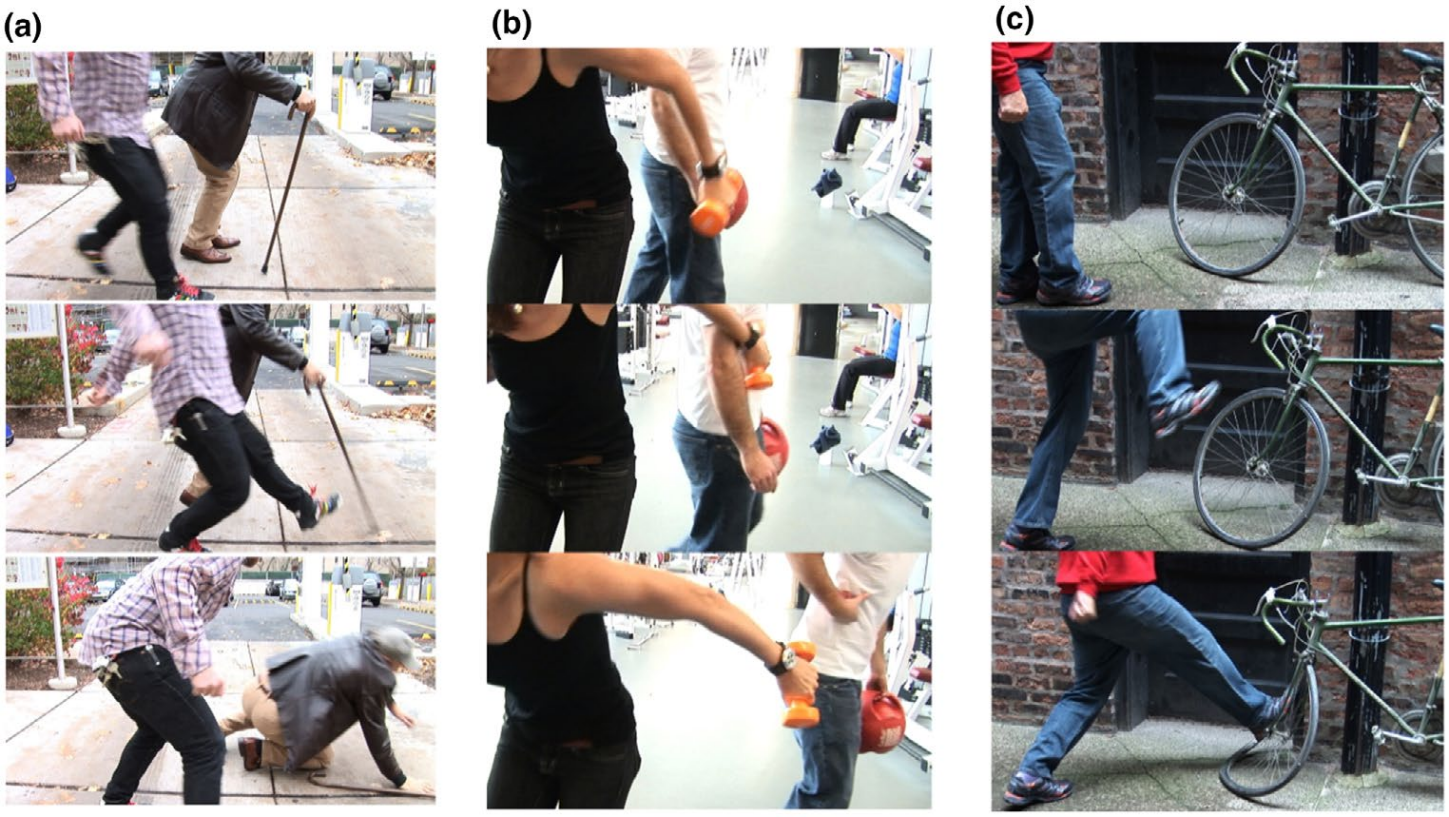
Examples of animated images in moral sensitivity task (Studies 1–2). Panel (a) illustrates a scenario depicting intentional harm to another person, representing the intentional condition. Panel (b) depicts a scenario portraying accidental harm to another person, representing the accidental condition. Lastly, panel (c) showcases a scenario demonstrating intentional harm to an object, serving as the control condition.

After giving their informed consent, participants completed a *Moral sensitivity task* (adapted from Decety et al., 2012), designed to measure their responses to different types of transgressions (see Figure 1 for examples). These included the following: (a) scenarios where one person purposefully causes harm to another person (intentional condition); (b) scenarios where one person unintentionally causes harm to another person (accidental condition); and (c) scenarios involving intentional harm directed at an object rather than a person (control condition). After each scenario, participants selected ‘Yes’ or ‘No’ to indicate whether the transgression was deliberate or unintentional (‘was the action done on purpose?’). Subsequently, they used visual analogue scales ranging from 0 to 100 to rate their concern for both the victim (‘how sorry do you feel for the injured person/damaged object?’) and the perpetrator (‘how sorry do you feel for the perpetrator?’), as well as their judgement of the perpetrator's meanness (‘how mean was the perpetrator?’) and appropriate punishment (‘how harshly would you punish the perpetrator?’).

The task comprised 2 initial practice trials, followed by 6 experimental trials (2 per condition, randomized across participants). The stimuli were created using 3 digital colour images, shown sequentially at durations of 500, 200 and 1000 ms to imply motion. Participants were blinded to the protagonists' faces to avoid facial bias, but the victim's emotional reaction and the perpetrator's intent remained inferable through body language. To reduce gender bias, the conditions included interactions across male‐to‐male, female‐to‐female, male‐to‐female and female‐to‐male pairings.

### Analysis strategy

First, we assessed the success of our manipulation by evaluating participants' accuracy in distinguishing between intentional and accidental conditions, with successful manipulation defined as correctly identifying intentionality in more than 50% of the trials. Next, we tested our hypothesis that participants would express greater concern for victims than for perpetrators by conducting a 2 (victim vs. perpetrator) × 3 (control vs. intentional vs. accidental) repeated‐measures ANOVA. Significant interactions were further explored using simple main effects analyses. Additionally, we examined differences in perceived perpetrator meanness and punishment ratings across conditions using one‐way repeated‐measures ANOVAs. For all analyses, follow‐up pairwise comparisons were conducted using a Holm‐Bonferroni correction, with an alpha level set at 0.05 and partial eta‐squared ($\eta_{p}^{2}$) reported as the effect size. We also reported 95% confidence intervals (95% CI) where applicable.

To test our hypotheses about how perceived perpetrator meanness and concern for perpetrators influence the relationship between concern for victims and punishment, we used structural equation modelling (SEM) with the lavaan package in *R* (Rosseel, 2012). We initially introduced the variables measuring concern for perpetrators and perceived meanness as mediators into separate models to investigate their mediation effects individually. Next, we examined the relative effects of these variables in a combined model. Significant interactions were probed using 1000 bootstrap samples from the original dataset and computed 95% bias‐corrected bootstrap confidence intervals (95% BCCI). Model fit was assessed using conventional indices, following recommended guidelines (Hu & Bentler, 1998; Schreiber et al., 2006). These included the chi‐square statistic (χ^2^), Root Mean Square Error of Approximation (RMSEA), Standardized Root Mean Square Residual (SRMR), Comparative Fit Index (CFI), and Tucker–Lewis Index (TLI). Acceptable fit was defined as RMSEA and SRMR values ≤ .08, and CFI and TLI values ≥ .90. A non‐significant χ^2^ (*p* > .05) was also considered indicative of good model fit, though interpreted with caution due to its sensitivity to sample size. Additionally, we report relative fit indices—including the Akaike Information Criterion (AIC), Bayesian Information Criterion (BIC), and the Sample‐Size Adjusted BIC (SABIC), with lower values indicating better fit—for comparisons.

### Results

#### Perceived intentionality

Participants accurately identified perpetrators' intentions in ~90–96% of cases involving intentional harm and in 75% of accidental harm scenarios, indicating that the experimental manipulation was successful.

#### Concern for victims versus perpetrators

**FIGURE 2:**
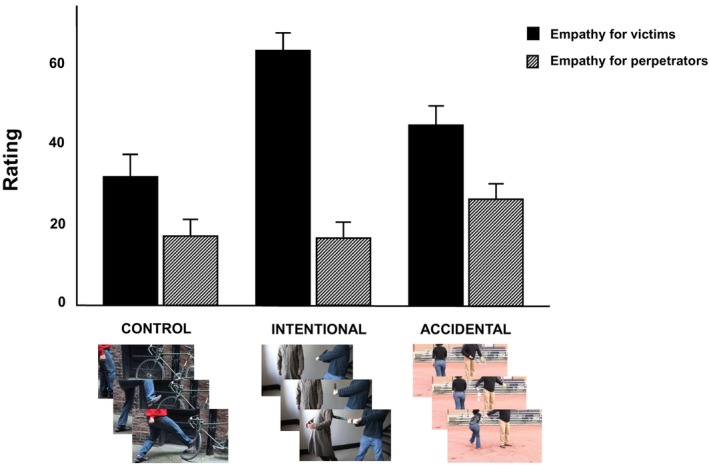
Empathic concern for victim vs perpetrator in each condition (Study 1). Error bars represent 95% confidence intervals. In all conditions, concern for victims was significantly greater than concern for perpetrators (control: *M* = 31.82, *SE* = 2.76, 95% CI [26.35, 37.29] vs. *M* = 17.21, *SE* = 2.01, 95% CI [13.20, 21.22]; intentional: *M* = 62.44, *SE* = 2.35, 95% CI [57.78, 67.10] vs. *M* = 16.66, *SE* = 2.04, 95% CI [12.61, 20.70]; accidental: *M* = 44.13, *SE* = 2.51, 95% CI [39.15, 49.11] vs. *M* = 26.34, *SE* = 2.04, 95% CI [22.30, 30.38]. All comparisons were significant at *p* < .001.

There was a significant interaction between expressed concern for victims and perpetrators across conditions, *F*(1,109) = 54.92, *p* < .001, $\eta_{p}^{2}$ = .33. As anticipated, participants expressed significantly more concern for victims in the intentional condition, *M* = 62.44, *SE* = 2.35, 95% CI [57.78, 67.10], compared to both the accidental, *M* = 44.13, *SE* = 2.51, 95% CI [39.15, 49.11] and the control, *M* = 31.82, *SE* = 2.76, 95% CI [26.35, 37.29], conditions. In contrast, participants expressed significantly less concern for perpetrators in both the intentional, *M* = 16.66, *SE* = 2.04, 95% CI [12.61, 20.70], and control, *M* = 17.21, *SE* = 2.02, 95% CI [13.20, 21.22], conditions compared to the accidental condition, *M* = 26.34, *SE* = 2.04, 95% CI [22.30, 30.38], whereas concern for perpetrators did not differ between intentional and control conditions, *p* = .793. Additional main effect analyses showed that expressed concern for victims was significantly higher than concern for perpetrators in each condition (see Figure 2 for these contrasts). All differences were significant at *p* < .001 (uncorrected), hence remaining significant at *p* < .05 after Holm–Bonferroni correction.

#### Judgements of perpetrator meanness and punishment

There was a significant difference in participants' ratings of both perceived perpetrator meanness, *F*(1,109) = 140.40, *p* < .001, $\eta_{p}^{2}$ = .56, and punishment severity, *F*(1,109) = 90.86, *p* < .001, $\eta_{p}^{2}$ = .45, across conditions. Participants rated the perpetrator as significantly meaner when the aggression was intentionally directed at another person, *M* = 68.96, *SE* = 2.03, 95% CI [64.94, 72.99], or at an object, *M* = 42.25, SE = 2.54, 95% CI [37.23, 47.28], compared to accidental harm to another person, *M* = 26.43, *SE* = 2.02, 95% CI [22.43, 30.44]. Similarly, participants supported significantly harsher punishments for intentional harm to both a person, *M* = 54.53, *SE* = 2.27, 95% CI [50.03, 59.04], and an object, *M* = 37.06, *SE* = 2.52, 95% CI [32.08, 42.05], than for unintentional harm, *M* = 21.38, SE =1.97, 95% CI [17.46, 25.29]. Post‐hoc pairwise comparisons revealed that these differences were significant at *p* < .05 after Holm–Bonferroni correction.

#### Direct and indirect effects of concern for victims on punishment

**FIGURE 3:**
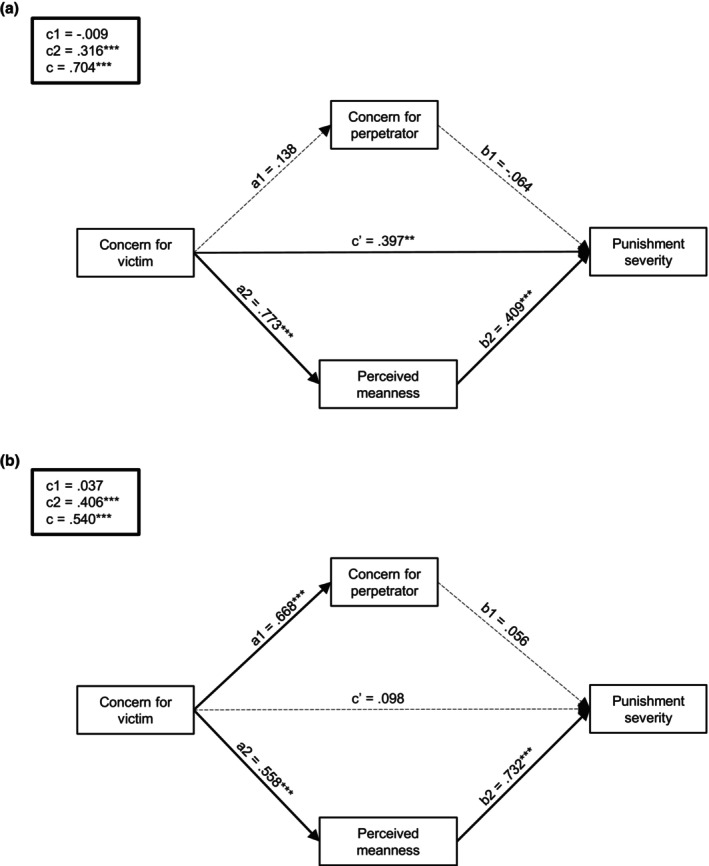
Path estimates in (a) intentional and (b) accidental conditions (Study 1). Mediation was assessed by examining the direct (c’) and indirect effects of interpersonal callousness on aggression through concern for the perpetrator (c1) and perceived perpetrator meanness (c2). The total effect (c) was the sum of the indirect effects through the mediators and the direct effect of the predictor. *p < .05, **p < .01, ***p < .001.

Table 1 provides direct and indirect effect estimates for each mediation model. Examination of individual mediation models revealed that concern for perpetrators did not significantly influence the relationship between concern for victims and punishment in neither condition. In contrast, perceived perpetrator meanness partially mediated the link between concern for victims and punishment in intentional scenarios, β = 0.32, *SE* = 0.09, 95% BCCI [0.13, 0.51], and fully mediated this relationship in accidental scenarios, β = 0.40, *SE* = 0.06, 95% BCCI [0.28, 0.54]. These effects remained when assessing concern for perpetrators and perceived meanness as mediators into combined models for each condition (see Figure 3). Fit indices indicated that both models were a good fit to our data, with relative fit indices suggesting better fit for the ‘accidental’ model (see Table S1).

**TABLE 1:** Direct and indirect effect estimates of mediation models in Study 1.

	Intentional			Accidental		
	*β*	*SE*	*BCCI*_*95%*_	*β*	*SE*	*BCCI*_*95%*_
*Model 1*						
Total effect	.704	.052	.**593, .799**	.544	.065	.**416, .667**
Direct effect	.713	.053	.**605, .808**	.653	.128	.**410, .894**
Indirect effect	−.010	.013	−.040, .017	−.109	.094	−.294, .072
*Model 2*						
Total effect	.704	.055	.**591, .804**	.544	.066	.**409, .667**
Direct effect	.386	.118	.**158, .640**	.143	.082	−.020, .300
Indirect effect	.317	.094	.**135, .508**	.401	.064	.**277, .536**
*Model 3*						
Total effect	.704	.056	.**595, .810**	.540	.066	.**411, .672**
Direct effect	.397	.121	.**169, .637**	.097	.141	−.152, .395
Indirect effect						
Via concern for perpetrator	−.009	.014	−.035, .024	.037	.075	−.123, .176
Via perceived meanness	.316	.095	.**132, .505**	.406	.067	.**278, .535**

*Note*: Model 1 = Estimates through individual mediation of concern for perpetrator; Model 2 = Estimates through individual mediation of perceived perpetrator meanness; Model 3 = Estimates through the combined mediation of concern for perpetrator and perceived perpetrator meanness. Reported direct effects are estimated after accounting for indirect effects. Significant estimates (BCCI_95%_ not including 0) are in bold.

### Discussion

The findings support previous research that people's judgements of perceived transgressions are significantly influenced by their perceptions of the perpetrator's intentions (Decety et al., 2012; Decety & Cowell, 2015; Mikula, 2003). Specifically, when harm is perceived as intentional, individuals tend to adopt harsher punitive attitudes towards the perpetrator (Decety et al., 2012). Furthermore, selectively applying empathy towards victims of aggression increased participants' tendency to impose harsher punishments on the aggressors. Notably, this effect was primarily mediated by participants' negative perceptions of the aggressor, aligning with existing literature that suggests that retaliatory responses to aggression are influenced by negative assessments of the aggressor (Carlsmith et al., 2002). In our study, this effect was particularly strong in scenarios of accidental harm, where the relationship between concern for victims and punishment severity was fully mediated by perceived perpetrator meanness. This finding implies that people's punitive attitudes against aggressors might be more influenced by their negative perceptions of the aggressor rather than their judgement of the aggression itself or their concern for the victim. In contrast, while participants also expressed lower concern for perpetrators, this did not seem to influence their decisions to punish, contradicting our initial expectations. These effects were further examined in a follow‐up study.

## STUDY 2

### Method

#### Participants

Even though the sample in Study 1 was large enough to detect the postulated effects, in this study we decided to almost triple our sample size to allow us to detect potentially small moderating effects of callous traits. A total of 310 participants (*M* = 22.75, *SD* = 1.75) were recruited via Prolific, a crowdsourcing platform for online research (www.prolific.co). Prolific allows researchers to recruit high‐quality, pre‐screened participants based on demographic or psychological criteria, and it ensures ethical compensation and participant anonymity (Palan & Schitter, 2018). All participants were based in the United Kingdom and received £7 as a compensation upon successful completion of the study. After giving their informed consent, participants reported their demographic information. More than half of the sample (60.6%) identified as White, with 16.5% of the remaining participants identifying as Asian/Asian British, 13.9% as Black and 9% coming from multiple or other ethnic groups. Next, participants performed the same experimental task described in Study 1, followed by survey questionnaires. This experiment was approved by the university's ethics committee as part of a larger online study.

#### Assessment of callous‐unemotional traits

We used the 24‐item *Inventory of Callous Unemotional Traits* (ICU; Frick et al., 2003) to assess callous‐unemotional traits. This questionnaire includes items denoting callousness (e.g. ‘The feelings of others are unimportant to me’), disregard for the consequences of one's actions on others (e.g. ‘I do not care who I hurt to get what I want’), and shallow affect (e.g. ‘I do not show my emotions to others’). Responses were provided on a 4‐point Likert scale from 0 (not at all true) to 3 (definitely true), and the summed score was computed for follow‐up analyses—excluding items 2 (‘What I think is right and wrong is different from what other people think’) and 10 (‘I do not let my feelings control me’) due to prior research indicating higher internal consistency after removing these items, along with their low item‐total correlations with the total ICU scale (Kimonis et al., 2008). After excluding these items, the remaining ICU items yielded high internal consistency, α = 0.82.

### Analysis

Initial analyses replicated the procedures described in Study 1. Additionally, we examined whether the relationship between concern for victims and punishment was moderated by levels of callous‐unemotional traits in both intentional and accidental conditions. To test these effects, we conducted multiple linear regression analyses that included the main effects of concern for victims and callous‐unemotional traits, as well as their interaction. Where the interaction term was significant, we used the interactions package in *R* to perform simple slopes analyses (Long & Long, 2021) estimating the effect of concern for victims on punishment at low, average, and high levels of callous‐unemotional traits. We also used the Johnson–Neyman technique to identify the range of callous‐unemotional trait values for which the relationship between concern for victims and punishment was statistically significant.

### Results

#### Perceived intentionality

Participants accurately identified intentional harm with a high success rate of 93.9%. In contrast, their accuracy in identifying unintentional harm was notably lower, at 55.5%. This discrepancy highlights the challenge participants faced in discerning the perpetrator's intentions in accidental scenarios. However, this accuracy still exceeded the pre‐established 50% threshold, indicating that (for the most part) participants were still able to identify intentionality in these conditions.

#### Concern for victims versus perpetrators

**FIGURE 4:**
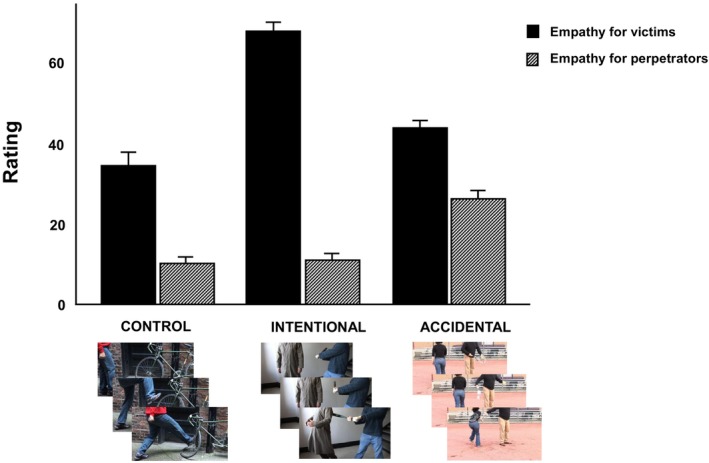
Empathic concern for victim vs perpetrator in each condition (Study 2). Error bars represent 95% confidence intervals. Across all conditions, concern for victims was significantly higher than concern for perpetrators (Control: *M* = 34.09, *SE* = 1.61, 95% CI [30.93, 37.25] vs *M* = 10.01, *SE* = 0.83, 95% CI [8.38, 11.65]; Intentional: *M* = 66.92, *SE* = 1.28, 95% CI [64.40, 69.44] vs *M* = 10.66, *SE* = 0.89, 95% CI [8.90, 12.41]; Accidental: *M* = 42.76, *SE* = 1.35, 95% CI [40.10, 45.42] vs *M* = 25.76, *SE* = 1.14, 95% CI [23.52, 28.00]). All comparisons were significant at *p* < .001***.

Consistent with our previous findings, there was a significant difference in participants' expressed concern for victims and perpetrators across conditions, *F*(1, 309) = 246.46, *p* < .001, $\eta_{p}^{2}$ = .44. However, simple main effects analyses indicated that participants expressed greater concern for victims when the transgression was intentional, *M* = 66.92, *SE* = 1.28, 95% CI [64.04, 69.44], than when it was accidental, *M* = 42.76, *SE* = 1.35, 95% CI [40.10, 45.42], or directed at an object, *M* = 34.09, *SE* = 1.61, 95% CI [30.93, 37.25]. In turn, concern for perpetrators was higher in the accidental harm condition, *M* = 24.76, *SE* = 1.14, CI 95% [23.52, 28.00.], than in both the intentional, *M* = 10.66, *SE* = 0.89, 95% CI [8.90, 12.41], and object harm, *M* = 10.01, *SE* = 0.83, 95% CI [8.38, 11.65], conditions. Further contrasts additionally showed that participants expressed significantly more concern for victims than for perpetrators in all conditions (main effects in Figure 4). All contrasts were significant at *p* < .05 after applying the Holm–Bonferroni correction, except for the difference between concern for perpetrators in the intentional versus control conditions, *p* = .472.

#### Moral judgements

There was a significant main effect of transgression type on both perceived meanness of the perpetrator, *F*(1, 309) = 511.47, *p* < .001, $\eta_{p}^{2}$ = .62, and punishment severity, *F*(1, 309) = 371.06, *p* < .001, $\eta_{p}^{2}$ = .55. Participants rated the perpetrator as significantly meaner when the harm was intentional, *M* = 73.61, *SE* = 1.05, 95% CI [71.54, 75.67], compared to harm directed at an object, *M* = 46.76, *SE* = 1.57, 95% CI [43.68, 49.84] or accidental harm to a person, *M* = 21.41, *SE* = 1.13, 95% CI [19.19, 23.63]. Similarly, witnessing intentional harm to another person led to harsher punishment decisions, *M* = 58.88, *SE* = 1.13, 95% CI [56.65, 61.11], than when harm accidentally caused to another person, *M* = 18.34, *SE* = 1.00, CI95% [16.38, 20.31], or directed at an objected, *M* = 37.64, *SE* = 1.41, 95% CI [34.87, 40.42]. All comparisons were significant at *p* < .001 (uncorrected), with significance retained after Holm–Bonferroni correction.

#### Mediating effects

Table 2 provides direct and indirect effect estimates for each mediation model. When evaluating each mediator in individual models, we found that perceived meanness partially mediated the relationship between concern for victims and punishment severity in both the intentional, β = 0.45, *SE* = 0.05, 95% BCCI [0.36, 0.57], and accidental conditions, β = 0.35, *SE* = 0.04, 95% BCCI [0.27, 0.43], indicating a positive effect. In contrast, concern for perpetrators emerged as a significant mediator in the accidental condition, β = −0.08, *SE* = 0.04, 95% BCCI [−0.15, −0.01], with data suggesting a partial negative effect.

**TABLE 2:** Direct and indirect effect estimates of mediation models in Study 2.

	Intentional			Accidental		
	*β*	*SE*	*BCCI*_*95%*_	*β*	*SE*	*BCCI*_*95%*_
*Model 1*						
Total effect	.614	.049	.**513, .704**	.460	.045	.**368, .549**
Direct effect	.614	.049	.**513, .704**	.542	.060	.**420, .652**
Indirect effect	−.000	.003	−.005, .005	−.082	.037	**−.154, −.007**
*Model 2*						
Total effect	.614	.048	.**510, .702**	.460	.046	.**372, .551**
Direct effect	.166	.075	.**022, .317**	.113	.040	.**033, .190**
Indirect effect	.447	.051	.**353, .557**	.347	.041	.**266, .426**
*Model 3*						
Total effect	.613	.046	.**515, .694**	.462	.047	.**370, .551**
Direct effect	.160	.070	.**019, .295**	.141	.055	.**033, .251**
Indirect effect						
via concern for perpetrator	−.000	.004	−.009, .007	−.025	.026	−.076, .026
via perceived meanness	.453	.050	.**364, .557**	.346	.041	.**269, .428**

*Note*: Model 1 = Estimates through individual mediation of concern for perpetrator; Model 2 = Estimates through individual mediation of perceived perpetrator meanness; Model 3 = Estimates through the combined mediation of concern for perpetrator and perceived perpetrator meanness. Reported direct effects are estimated after accounting for indirect effects. Significant estimates (95% BCCI not including 0) are in bold.

**FIGURE 5:**
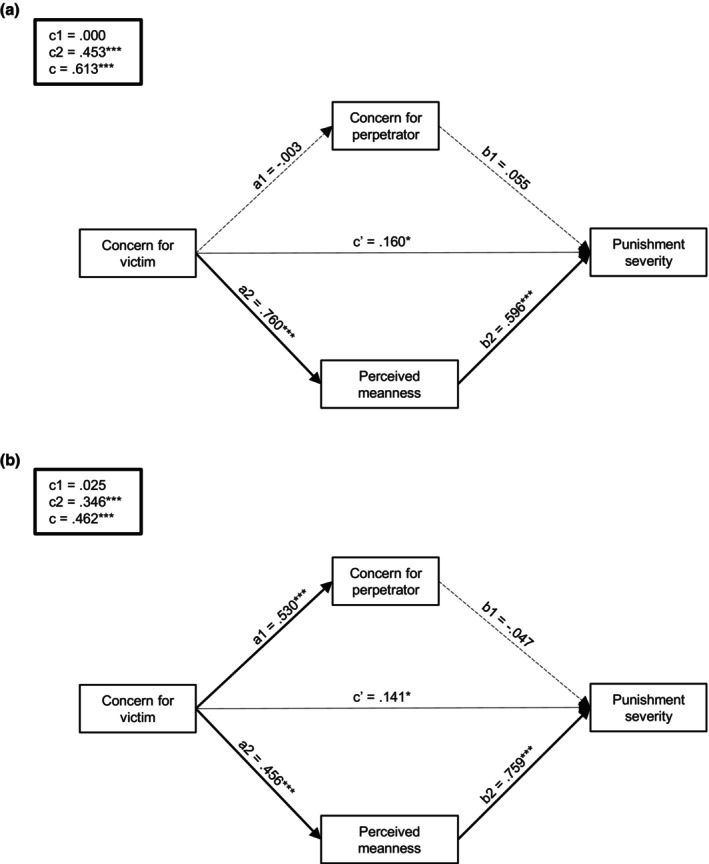
Path estimates in (a) intentional and (b) accidental conditions (Study 2). Mediation was assessed by examining the direct (c’) and indirect effects of interpersonal callousness on aggression through concern for perpetrator (c1) and perceived perpetrator meanness (c2). The total effect (c) was the sum of the indirect effects through the mediators and the direct effect of the predictor. *p< .05, **p< .01, ***p< .001.

Nevertheless, when introducing both mediators into a combined model, only the effects of perceived meanness remained significant. In line with Study 1, perceived perpetrator meanness significantly mediated the effects of empathy on punishment severity in both conditions. In this case, however, we observed a partial mediation effect in the accidental condition, β = 0.35, *SE* = 0.04, 95% BCCI [0.27, 0.43], and a full mediation effect in the intentional condition, β = 0.45, *SE* = 0.05, 95% BCCI [0.36, 0.56] (see Figure 5 for an illustration). Fit indices overall indicated good fit for both models, with the ‘intentional’ model showing better fit. These values are reported in Table S2.

#### Effects of callous‐unemotional traits

Lastly, we sought to explore the links between concern for victims and punishment for perpetrators through the moderation of callous‐unemotional traits. As anticipated, higher concern for victims was associated with harsher punishment ratings in both the intentional, β = 0.80, *SE* = 0.11, *p* < .001, and accidental, β = 0.27, *SE* = 0.10, *p* = .005, conditions. However, in the intentional harm condition, the effect of victim concern on punishment severity weakened at higher levels of callous‐unemotional traits, β = −0.01, *SE* = 0.01, *p* = .033. Interestingly, participants with more callous‐unemotional traits also supported more severe punishment overall, β = 1.09, *SE* = 0.32, *p* = .001.

**FIGURE 6:**
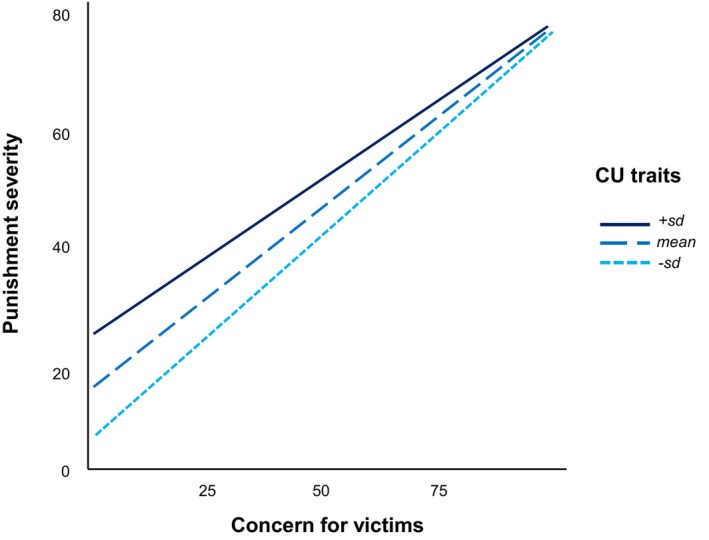
Link between punishment severity and victim bias in the intentional condition (Study 2). Effects of empathy on punishment severity at higher (+1 SD), mean, and lower (−1 SD) levels of callous‐unemotional traits (CU traits).

Simple slopes analyses (Figure 6) revealed that the relationship between victim concern and punishment remained significant across different levels of callous‐unemotional traits (all *p*s < .001). In contrast, in the accidental harm condition, callous‐unemotional traits did not significantly predict punishment severity, β = 0.22, *SE* = 0.21, *p* = .314, nor did they moderate the effect of concern for victims, β = 0.00, *SE* = 0.00, *p* = .291.

### Discussion

The outcomes of this study largely replicated Study 1, demonstrating the robustness of our observations. Expanding on this, the current study further indicates that the impact of empathy on participants' punishment decisions for perceived aggression diminishes at higher levels of callous‐unemotional traits. This observation supports our hypothesis that callous‐unemotional traits attenuate the empathic bias in punitive decision‐making.

On the other hand, however, participants with higher levels of callous‐unemotional traits were also more prone to advocating for harsher penalties against aggressors. This finding challenges the notion that callous‐unemotional traits might promote judicial impartiality. Instead, it suggests that individuals with these traits may be more predisposed to endorsing harsher punitive measures—which is consistent with links between callous‐unemotional traits and aggression highlighted in previous research (Brugman et al., 2017; Camara et al., 2025; Frick et al., 2003; Ritchie et al., 2022). The broader implications of these findings are discussed in greater detail in the following section.

## GENERAL DISCUSSION

Our findings align with existing research on how evaluations of transgressions are largely influenced by perceptions of the perpetrator's intentions. Consistent with the Attribution of Blame model (Shaver & Shaver, 1985), which proposes that perceived responsibility for norm violations drives moral evaluations, our results confirmed that when harm is perceived as intentional—a condition under which blame is more readily assigned—participants were more likely to endorse harsher punishments. This reflects the inherent subjectivity in moral judgements, showing that the same act can be punished differently depending on the perceived intent behind it. Research additionally shows that intentional aggressions are more strongly condemned than accidental ones (Baez et al., 2014; Decety et al., 2012; Young & Saxe, 2008). Our study extends this literature by providing preliminary insights into how such evaluations relate to evaluative judgements and empathic responses towards the perpetrator.

Partially in line with our initial hypotheses, we found that participants expressed greater concern for victims than for aggressors, although their expressed concern for the perpetrator did not seem to directly influence decisions to punish. Rather, perceived meanness of the perpetrator emerged as a significant mediator in the relationship between concern for victims and punishment severity, suggesting that negative appraisals may play a more central role in justice‐related decisions than empathic concern alone. This is consistent with previous research indicating that biases in punitive decision‐making can be largely influenced by people's perceptions of the parties involved (e.g. Bastian et al., 2013; Viki et al., 2012). In fact, it is plausible that perceived perpetrator meanness influences empathic responses to the aggressor as well. That is, seeing the perpetrator as ‘mean’ may serve to justify the withholding of empathy and reinforce punishment (e.g. Bastian et al., 2013; Harris & Rice, 2006; Osofsky et al., 2005). In this view, one potential avenue for follow‐up research could be to further examine whether the link between concern for perpetrators and punishment severity might be mediated by perceived perpetrator meanness.

Furthermore, the current work contributes to the literature by examining how individual differences in callous‐unemotional traits influence these empathic processes and their relationship to punitive attitudes, opening new avenues for further exploration. Our findings indicate that individuals with more callous‐unemotional traits exhibit lower concern for victims and a weaker link between victim‐focused empathy and punishment yet still tend to favour harsher punishments overall. Interestingly, this pattern aligns with findings from studies of patients with the behavioural variant of frontotemporal dementia (bvFTD)—a neurodegenerative condition marked by poor affect, diminished empathy and impaired social cognition (Piguet et al., 2011; Rascovsky et al., 2011). Patients with bvFTD have been shown to rate punishments as more severe, regardless of whether harm was inflicted intentionally or accidentally (Baez et al., 2014), a pattern that mirrors the reduced concern and elevated punishment ratings observed at higher levels of callous‐unemotional traits in our study. These convergences support the view that traits associated with emotional detachment and reduced affective resonance may reduce the impact of perceived intentionality on moral evaluations.

Supporting this interpretation, our data further showed that in scenarios involving accidental harm, callous‐unemotional traits did not moderate the relationship between concern for victims and punishment, even though they were correlated with increased punishment severity in these cases. This could suggest that punitive decisions among individuals with higher levels of callous‐unemotional traits may be less guided by empathic sensitivity or moral evaluation, and more by a rigid or indiscriminate orientation towards punishment—consistent with prior research indicating that callous‐unemotional traits confer unique risks for more instrumental or severe forms of aggression (e.g. Brugman et al., 2017; Camara et al., 2025; Frick et al., 2003). These possibilities gain special relevance in legal and forensic contexts, where individual traits may predispose certain decision‐makers (e.g. jurors) to punitive bias, regardless of contextual factors like intent or remorse. They also underscore the importance of considering personality‐driven variations in justice motivation, which may help inform targeted interventions or training programs aimed at promoting fairness and reducing bias in evaluative judgements.

### Limitations

Altogether, our findings highlight the nuanced role of perceptions of both aggression and the aggressor in shaping punishment decisions, suggesting that punitive attitudes are influenced by a combination of empathy, cognitive evaluation and context. However, these interpretations should be approached with caution due to the study limitations. One key issue lies in the underlying assumption that emotion and partiality are intrinsically linked, suggesting that impartiality is more unemotional or rational. While the ideal of judicial dispassion is deeply ingrained in legal theory and popular opinion, recent arguments (e.g. Maroney, 2024) suggest that emotions can be effectively managed and do not inevitably lead to bias or partiality. Relatedly, our analysis of fairness and justice has primarily focused on emotional influences, yet these concepts encompass a broader range of factors. For instance, elements such as social identity, familiarity and other contextual variables might also predispose individuals to certain biases in their moral judgements (e.g. Abbink & Harris, 2019; Schiller et al., 2014). Consequently, the scope of our analysis is limited, and future research should consider these interactive factors to provide a more comprehensive understanding of the dynamics underlying punitive decision‐making.

In addition, the lack of a consistent definition of empathy poses challenges in generalizing our findings to its various dimensions. As noted by Brown (2012, p. 386): ‘Because the definitions and understandings of empathy we work with are generally positioned in the realm of the abstract, then empathy ‘in the field’ and ‘on the ground’ may require alternative dimensions in its articulation’. In other words, given the multifaceted nature of empathy, reliance on one conceptualization may risk oversimplifying the role empathy plays on behavioural outcomes, as it neglects the contextual and situational factors that influence how empathy is expressed in everyday social behaviour. As such, it is essential to clarify which aspect of empathy is under investigation—be it cognitive or affective—and to consider how these facets manifest differently across various social contexts (Vachon & Lynam, 2016). This distinction is crucial because each facet of empathy might interact with moral judgement and punitive decision‐making in unique ways. Although fully resolving these definitional ambiguities is beyond the scope of our current work, it highlights an important consideration that should be explored in future studies looking into the role of empathy in punishment.

Moreover, the use of animated images to elicit empathy is another key limitation in the current research. While animated stimuli may offer a more dynamic representation compared to static images (Decety et al., 2012), they cannot fully capture the complexity and nuance of real‐life situations. In everyday scenarios, moral judgements are often influenced by the contextual details surrounding the actions of the perpetrator and victim (Jin & Peng, 2021). By omitting such contextual information, our study may have limited the scope of participants' responses, preventing a more comprehensive understanding of the factors that influence punitive decision‐making. Future research that incorporates more detailed background context may help capture a broader range of reactions and provide a more accurate reflection of real‐world moral judgements.

Finally, while our sample was designed to examine patterns in a normative population, our exploration of callous‐unemotional traits would benefit from studying a sample with a broader or more pronounced distribution of these traits. Individuals with higher levels of callous‐unemotional traits—such as those classified as ‘psychopaths’—may exhibit more distinct patterns of empathy and punishment, offering a deeper understanding of their role in justice‐related decisions. This approach could provide a more nuanced account of how specific socio‐affective traits shape punitive attitudes and behaviours.

## CONCLUSION

Overall, the common thread across our findings is that people's punishment decision‐making is influenced by their perceptions of both the aggression and the aggressor, although the latter seems to have a greater effect. While punishment severity significantly increased for intentional transgressions, negative perceptions of the perpetrator influenced punishment regardless of the intentionality of the transgression itself. This suggests that punitive responses are not solely guided by principles of justice—such as proportionality or the need for correction—but are also susceptible to biases rooted in how we perceive those we judge. Notably, the tendency for individuals with more callous‐unemotional traits to endorse harsher punishment regardless of perceived intentionality suggests that punishment may also reflect something deeper in an individual's moral compass, such as a greater predisposition to engage in interpersonal harm (especially if there is no motivation for retribution or vengeance). These reflections provide a basis for future research to explore the broader implications of callous‐unemotional traits in moral judgement and punitive decision‐making.

## AUTHOR CONTRIBUTIONS

**Célia F. Camara:** Conceptualization; investigation; writing – original draft; methodology; formal analysis; project administration; visualization; data curation. **Alejandra Sel:** Funding acquisition; supervision; writing – review and editing. **Paul H. P. Hanel:** Conceptualization; validation; writing – review and editing; supervision; project administration.

## FUNDING INFORMATION

This research was supported by the Academy of Medical Sciences Springboard Award (SBF008\1113) and the Essex ESNEFT Psychological Research Unit for Behaviour, Health and Wellbeing (RCP15313), awarded to Alejandra Sel. The funders had no role in the study design, data collection, analysis, interpretation, manuscript preparation, or the decision to submit for publication.

## CONFLICT OF INTEREST STATEMENT

The authors report no conflicts of interest related to this work.

## Supporting information


Table S1.

## Data Availability

The data that support the findings of this study are openly available in Open science framework at https://osf.io/bfmnt/?view_only=180a8ea9aea140b69f97af6b4b238ffc.
