# Supplementary material for: When empathy leads to aggression: The effects of empathy on punitive attitudes towards aggressors
Source: Br J Soc Psychol. 2025 May 23;64(3):e12907. doi: 10.1111/bjso.12907 (PMC12102588; doi:10.1111/bjso.12907)
Supplement: Supplementary file 1 — Table S1. [file BJSO-64-0-s001.docx]

**Supplementary Information**

When empathy leads to aggression: The effects of empathy on punitive attitudes towards aggressors

Célia F Camara*, Alejandra Sel, Paul HP Hanel

*Department of Psychology, University of Essex*

**^*^Corresponding author:**

Célia F Camara

c.camaraperezvera@essex.ac.uk

camaracelia@outlook.com

| **Table S1**  *Fit Indices (Study 1)* | | |
| --- | --- | --- |
|  | **Intentional** | **Accidental** |
| **Chi** | .001 | .001 |
| **CFI** | 1.000 | .968 |
| **TL1** | 1.031 | .805 |
| **RMSEA** | .000 | .257 |
| **SRMR** | .004 | .065 |
| **AIC** | 2799.757 | 2739.329 |
| **BIC** | 2821.361 | 2760.932 |
| **SABIC** | 2796.081 | 2735.652 |
| *Note*. Model fit comparisons for the combined mediation of concern for perpetrators and perceived meanness in both intentional and accidental conditions. | | |

| **Table S2**  *Fit Indices (Study 2)* | | |
| --- | --- | --- |
|  | **Intentional** | **Accidental** |
| **Chi** | .079 | .016 |
| **CFI** | .996 | .991 |
| **TL1** | .975 | .945 |
| **RMSEA** | .082 | .124 |
| **SRMR** | .024 | .041 |
| **AIC** | 7524.84 | 7618.70 |
| **BIC** | 7554.73 | 7648.60 |
| **SABIC** | 7529.36 | 7623.22 |
| *Note*. Model fit comparisons for the combined mediation of concern for perpetrators and perceived meanness in both intentional and accidental conditions. | | |
